# How physical activity and passion color the passage of time: A response with ultra-trail runners

**DOI:** 10.3389/fpsyg.2022.934308

**Published:** 2023-01-06

**Authors:** Quentin Hallez, Marine Paucsik, Guillaume Tachon, Rebecca Shankland, Fanny Marteau-Chasserieau, Mathilde Plard

**Affiliations:** ^1^DIPHE, Institut de Psychologie, Univ. Lumière Lyon 2, Lyon, France; ^2^Univ. Savoie Mont Blanc, Univ. Grenoble Alpes, Grenoble, France; ^3^Ecole de Psychologues Praticiens, Paris, France; ^4^Institut Universitaire de France, Paris, France; ^5^ESO, UMR 6590, Univ Angers, Angers, France

**Keywords:** time perception, passage of time, physical activity, ultra-trail, harmonious passion, obsessive passion

## Abstract

The aim of this study was to replicate the results of a lengthening effect caused by physical activity already observed in duration length judgment, using the time passage judgment measure, while exploring the effects of passion types (obsessive vs. harmonious) on time perception. A total of 378 ultra-trail runners responded to an online questionnaire in which the type of passion and the passage of time (PoT) judgments associated with both an ultra-trail context and a non-trail daily context were collected. The results showed that participants systematically judged the time as being dilated in a situation of sports practice, thus extending the results obtained in interval duration judgment studies. This study also showed an influence of the type of passion: higher levels of harmonious passion were related to greater feelings of time dilation, while higher levels of obsessive passion were related to greater feelings of both time contraction and time dilation. Results are discussed in light of the two major factors that influence the PoT referenced in the literature, namely, attention and happiness level.

## Time perception and physical activity

The terminology of “ultra-trail” is a trademark registered by the organizers of the Ultra-Trail du Mont-Blanc (UTMB), which is a very important athletic event consisting of seven different foot races that cross three countries, namely, France, Italy, and Switzerland. Ultra-trail is now commonly used generically to describe very long-distance trails, over 80 km (50 miles). Over the past years, we have witnessed a massification of the practice. According to the UTBM, 722 runners registered for their races in 2003 (Duquenne, 2021) compared to 32,000 in 2020 (L'Équipe, 2020). Therefore, this represents an increase of 4,332% participation in 17 years. One could, therefore, wonder what are the motivations of these more numerous individuals, immersing themselves for multiple hours in this physical activity, within an environment subject to hazards, climatic, topographical, and temporal constraints (Travert et al., 2019). But is the perception of time in a trail situation only comparable with the perception of time outside of practice? This study aims to answer this question within the field of the passage of time judgment (PoT) while investigating the influence of the type of passion that the participant has for ultra-trail on this measure.

When referring to time perception, this necessarily implies the existence of two distinct times, namely, (1) an objective and universal physical time, the flows of which are linear in a constant rate and (2) a subjective and psychological time, the flows of which can be modulated depending on the individuals. Although the brain measuring psychological time is a real-time processing system, it sometimes does not match the physical time measured by our time measurement tools. On a daily basis, our brain can deceive us depending on contexts, by underestimating or overestimating the physical time elapsed, thus allowing for the transformation of the same physical duration into a fraction of a second or an eternity, subjectively speaking. Researchers distinguish two main subjective time experiences, namely, interval length judgments and subjective PoT judgments, as they have been suggested to rely on different mechanisms (Droit-Volet and Wearden, 2016). Perception of interval length concerns the subjective evaluation of a certain duration, while subjective PoT relies on the perceived speed of the time course which is typically assessed *via* the first-person reports on the individual's own feelings on time passage (Wearden, 2005; Friedman and Jansse, 2010).

To the best of our knowledge, no study has investigated the topic of physical activity and time perception under the prism of subjective PoT judgments. Yet, several studies have been conducted on interval length judgments and systematically showed an overestimation of time (i.e., time is perceived as longer than it really is) as a function of the intensity of the exercise: the more intense, the further the dilation of time (for a review, refer to Behm and Carter, 2020). This higher arousal has also been detected for long durations (verified up to 5 min in Edwards and McCormick, 2017). It has been proposed that as the intensity of physical effort grows, sensory awareness increases due to a state of hyperarousal. This state would be caused by catecholamine, which is a hormone produced by the body during psychological or physical stress (Jansen et al., 1995; Goldstein, 2003). This effect of acute sensory awareness would also be maximized by the participant's suffering, preventing them from being able to remove their attention from the present moment (Edwards and Polman, 2013; Tamm et al., 2014, 2015). The fact that this effect was detected over long intervals allows us to predict that the participants should report a feeling of dilation of time in their judgments of the PoT. Indeed, an important study by Droit-Volet and Trahanias, 2017 demonstrated that length judgment associated with long durations (> 60 s) was highly correlated with PoT judgments. It is important to note that this positive correlation between length judgment and PoT judgment was only observed for durations longer than 60 s, while no relation was observed between the two abilities for shorter durations (Droit-Volet and Wearden, 2016). According to Droit-Volet and Trahanias (2017), this could be due to the fact that PoT and length judgment, when judging long durations, would involve memory reconstruction processes that rely on non-temporal information stored in the memory. In other words, it would rely on the amount of internal and external contextual changes experienced (Martinelli and Droit-Volet, 2022). This is well supported by the model proposed by Roseboom et al. that demonstrated, using an artificial neural network, that time perception might be accomplished, based on non-temporal perceptual classification processes (Roseboom et al., 2019). According to the model, the more there is non-temporal perceptual classification, the more time is perceived as long.

It is noteworthy that there could be another alternative. When individuals experience intense flow—flow being considered the expression of optimal psychological experience (Csikszentmihalyi and Csikzentmihaly, 1990)—they sometimes declare finding themselves in timelessness. In such situations, there is no experience of time dilation or contraction, since time no longer exists (Csikszentmihalyi, 1988). This is important because this phenomenon of flow during sports has been reported many times in the literature (for a systematic review, refer to Swann et al., 2012). Timelessness might happen under extreme situations, when the task is too difficult, or during highly intense emotions. This phenomenon has already been reported in deep states of meditation experience when performed by meditation experts (Droit-Volet and Dambrun, 2019), as well as in car accidents (Arstila, 2012) or near-death experiences (Martial et al., 2017). Thus, some runners might have difficulties answering questions about making PoT judgments paired with sports.

## The dualistic model of passion

Passion can be defined as “A strong inclination toward a specific object, activity, concept or person that one loves (or at least strongly likes), highly values, invests time and energy in on a regular basis, and that is part of one's identity” (Vallerand and Verner-Filion, 2020; p 207). Over the past 15 years, Vallerand and his colleagues (Vallerand, 2001, 2008, 2010, 2015, 2019; Vallerand and Houlfort, 2003; Vallerand et al., 2003) developed the dualistic model of passion to fill the void in the psychological literature on this construct and to address the issues of the nature, determinants, and outcomes of passion. This dualistic view opposes obsessive passion with harmonious passion. Obsessive passion can clearly be illustrated by this citation of Marie-Henri Beyle: “Every true passion thinks only of itself” (Stendhal, 1830; p. 146) as it is characterized by the internal pressure/compulsion of individuals to practice an activity. The lack of control experienced by individuals over the activity leads them to be dependent on the activity (Vallerand and Houlfort, 2003). In the same way, as a person becomes dependent on an illicit substance, the activity takes more space in the individual's daily life and identity, which in turn generates conflicts between passion and other life areas. This in turn creates difficulties in turning away from the activity and generates persistence to engage in the passionate activity even when the circumstances surrounding activity engagement make it ill-advised or counterproductive. For example, people with an obsessive passion are more likely to engage in their activity when injured (Rip et al., 2006). Conversely, harmonious passion, as its name implies, is in harmony or balance with other life areas. This type of passion is much healthier as individuals have control over the activity and are able to regulate their engagements (e.g., stop the practice while injured). The use of this dualistic view in sports has been clearly documented and validated (for a review, refer to Vallerand and Verner-Filion, 2020).

Researchers showed that obsessive and harmonious passion are, respectively, negatively and positively linked to well-being (Vallerand et al., 2007). Indeed, the more an individual has an obsessive passion, the further they ruminate about their passionate activity while engaging in another activity, which, ultimately, would lead to lower levels of well-being (Carpentier et al., 2012). Interestingly, it has been recently demonstrated that negative feelings such as frustration cause slowing-down effects on the PoT (i.e., time passes slower) (Tipples, 2018). The negative feelings that would emerge in obsessive runners when they are not engaging in their obsessive passionate activity could, thus, create an experience of time slowing down. Conversely, it has been demonstrated that harmonious passion was a positive predictor of life satisfaction and subjective happiness, which could lead to greater levels of well-being (Yukhymenko-Lescroart, 2019). In the field of PoT, it has been clearly demonstrated that one's level of happiness has an effect on their awareness of the PoT. In numerous studies, participants systematically report that time flies when they are happy and that it drags on when they are sad (Droit-Volet and Wearden, 2015, 2016; Droit-Volet and Trahanias, 2017; Droit-Volet and Dambrun, 2019; Droit-Volet et al., 2021). In a recent study, researchers experimentally investigated PoT judgments in a laboratory and demonstrated that the valence associated with a stimulus has a critical effect on PoT. The speed of the PoT was judged to increase with the positive valence of the emotional stimuli, regardless of their presentation duration (Martinelli and Droit-Volet, 2022).

The aim of this study was thus 2-fold. First, we focused on the subjective sensation of the PoT that ultra-trail runners report experiencing in their sports practices and compare these sensations with those of daily life, outside of sports practice. Second, we analyzed how the type of passion that the participant has for ultra-trail impacts their temporal representations. Our hypotheses were, thus, as follows: (1) ultra-trail practice should lead to further feelings of temporal distortion and, more specifically, (2) temporal dilation of time compared to everyday life activities, outside of sports practice. We also expected that (3) higher feelings of dilated time would be associated with higher scores of obsessive passion and that (4) higher feelings of contracted time would be paired with higher scores of harmonious passion, independently of the activity.

## Methods

### Participants

The sample of this study was made up of 378 adult participants (*M* = 42.42, *SD* = 9.82, [18–89 years], 84 women), recruited online *via* an email sent to all the Ultra-Trail Mont Blanc (UTMB) subscribers. The participants were predominantly European (*N* = 351, 92.9%) of whom 237 were French. In addition, there were 11 (2.9%) North Americans and 2 (0.5%) South Americans, 7 (1.9%) Asians, 5 (1.3%) Oceanians, and 2 (0.5%) South Africans. All the participants had practiced ultra-trail running for at least 1 year (*M*_practice_ = 13.78 years, *SD*_practice_ = 10.00 [1–62 years]), and their level of practice ranged from beginners to professionals as stated by the ITRA score which is reference ranking in trail running (N^unclassified^ = 135; N^classified^ = 243, *M* = 519.85, *SD* = 88.11, [290; 790]). Participants provided informed consent prior to completing the experiment, which was in accordance with the 1964 Declaration of Helsinki.

### Materials

The survey was conducted using the Qualtrics^®^ electronic survey software, which recorded participants' responses and delivered the stimuli. The passion scale (Vallerand et al., 2003; Marsh et al., 2013) was used to measure the harmonious passion and the obsessive passion of athletes toward running. These two subscales are measured on a Likert-type scale ranging from 1 (Not agree at all) to 7 (Very strongly agree). There are six items measuring harmonious passion (i.e., *This sport is in harmony with the other activities in my life*, α = 0.76) and six items measuring obsessive passion (i.e*., I have almost an obsessive feeling for this sport*, α = 0.83). The harmonious and obsessive passion scores, therefore, consist of the addition of these six respective items. The scores can, therefore, range from 6 to 42. In addition, five questions about the PoT were asked on a 100-point self-evaluation scale ranging from 0 (not at all) to 100 (absolutely), both in the context of general life and ultra-trail practicing. In this set of PoT questions, two of them were related to feelings of time distortions (i.e., *Time seems to be changing* (either slowing down or speeding up); *Time seems to flow differently than usual)*. Because the two items showed a satisfactory positive correlation (*r* = 0.67, *p* < 0.001), we averaged them to create a single item that we called “*Feeling of time distortions.”* The last three questions were related to overestimation and underestimation of time (i.e., *I feel like time is going fast*; *I feel like the amount of elapsed time is short; I feel like time stands still*). Yet, the 3 latter items did not show satisfactory internal consistency and, therefore, were treated separately (α = 0.57).

### Procedure

Participants were first invited to answer demographic questions (gender and age), as well as questions related to their practice of ultra-trail (since when they practice, number of training sessions per week, ITRA score). It should be noted that we had no assumptions about these elements. These questions simply allowed us to appreciate the characteristics of our sample. The presentation ordering of the 3 questionnaires (e.g., the passion scale; feeling of PoT—for everyday life, outside of sports activities; feeling of PoT—ultra-trail running) were randomized across participants.

## Results

Data were analyzed using SPSS version 26 (IBM SPSS, 2019). Table 1 shows the descriptive statistics associated with each of the experimental variables. A harmonious score was deleted from the database because it was detected as an outlier score (harmonious passion = 8). This deletion did not change the results but allowed us to run repeated measures ANOVAs while respecting the conditions related to these analyses. As illustrated by the table below, the values of skewness and kurtosis did not exceed the +2 to −2 range threshold (Kim, 2013; Van Zyl et al., 2021), indicating that the variables did not violate the assumptions of multivariate normality (normal distribution). Furthermore, all Mauchly's sphericity tests were non-significant (*p* > 0.05); the variances of the difference between all combinations of the within- and between-group factors were equal (i.e., to test for an increase in Type 1 error).

**Table 1:** Descriptive statistics.

	***N***	**Mean**	**S.D**.	**[Min; Max]**	**Skewness**	**Kurtosis**
Harmonious passion	377	33.46	4.10	[21; 42]	−0.34	0.07
Obsessive passion	378	19.81	7.46	[6; 42]	0.26	−0.57
**Everyday life (outside of sports)**						
Feeling of time distortions	378	50.35	28.27	[0; 100]	−0.19	−0.93
Time goes fast	378	64.82	29.00	[0; 100]	−0.65	−0.63
Elapsed time is small	378	32.71	28.57	[0; 100]	0.72	−0.58
Time stands still	378	30.28	29.36	[0; 100]	0.83	−0.51
**Trail running**						
Feeling of time distortions	378	71.68	24.25	[0; 100]	−0.97	0.32
Time goes fast	378	60.26	29.89	[0; 100]	−0.25	−0.83
Elapsed time is small	378	44.13	30.58	[0; 100]	−0.48	−1.16
Time stands still	378	55.69	32.67	[0; 100]	0.19	−1.30

N represents the total number of observations (individuals); S.D. refers to Standards Deviation; [Min; Max] refers to the minimum and maximum data observed in our sample.

### Feeling of time distortions

First, in order to analyze the general influence of the context independently of the type of passion, a repeated measures ANOVA was performed on the feeling of time distortion reported by participants, with the within-subject variable of context (everyday life vs. trail running). The ANOVA revealed a strong influence of the context, *F*_(1,377)_ = 162.39, *p* < 0.001; η^2^_p_ = 0.30; 1-β = 1.0, with higher time distortions reported in the trail-running context (*M* = 71.68, *SD* = 24.25) compared to an everyday life situation, outside of sports practices (*M* = 50.35, *SD* = 28.27).

The same model was performed by integrating the 2-passion type as co-variables. This time, the main effect of context was not significant, *F*_(1,377)_ = 1.03, *p* = 0.31. However, there was a significant harmonious passion × context interaction effect, *F*_(1,377)_ = 10.67, *p* = 0.001; η^2^_p_ = 0.03; 1-β = 0.90, as well as an obsessive passion × context interaction effect, *F*_(1,377)_ = 5.28, *p* = 0.02; η^2^_p_ = 0.01; 1-β = 0.63. Additional regression analysis of the harmonious passion scores on the difference between everyday life (without physical activity) and trail-running feeling of time distortion revealed that the higher the harmonious score, the higher the temporal distortion felt by participants of everyday life (without physical activity) compared to trail-running context (*t* = −2.74, β = −0.14, *SE* = 0.41, *p* = 0.006). The regression performed with obsessive passion scores did not reach significance on its own (*t* = 1.40, β = 07, *SE* = 0.22, *p* = 0.16), but suggested that the effects detected in the ANOVA go in the opposite way, with higher obsessive scores being linked to a reduced distortion effect from everyday life activities to trail-running context. Interestingly, the ANOVA also revealed a main effect of obsessive passion, *F*_(1,377)_ = 104.81, *p* < 0.001; η^2^_p_ = 0.04; 1-β = 0.97. Indeed, a linear regression of obsessive scores on the mean felt temporal distortion revealed that the higher the scores of obsessive passion, the higher the feelings of temporal distortion, regardless of the context. The main effect of harmonious passion did not reach significance, *F*_(1,377)_ = 1.11, *p* = 0.29. In sum, the distortions observed, which are more important in sports practice than in daily life situations, depend on the type of passion. The higher the score of harmonious passion, the greater the magnitude of the difference between the contexts. Conversely, the magnitude of the difference will decrease as the obsessive passion scores increase. Finally, the obsessive passion score directly predicts time distortion, independently of the context (e.g., the higher the obsessive passion score, the higher the subjective feeling of time distortion).

### Overestimation and underestimation of time

Thus, we have focused on analyzing the temporal distortion reported by the participants, but we still do not know in which direction this distortion can be observed (e.g., contraction or dilation?). Although they did not show a sufficient correlation between them to be related, the two overestimation indices (e.g., (1) time goes fast and (2) the elapsed time is short) showed strictly similar results. As a consequence, we will only present the results obtained on one of them (“time goes fast” was randomly selected). An ANOVA was performed on this variable within the variable of context (everyday life vs. trail running). Once again, the ANOVA revealed a main effect of the context, *F*_(1,377)_ = 5.07, *p* = 0.025; η^2^_p_ = 0.01; 1-β = 0.61, with a subjective feeling of acceleration of time more marked in everyday life (M = 64.82, SD = 29.00) than in sports practice (M = 60.26, SD = 29.89).

These results are in line with the measurement of time underestimation (e.g., time stands still), since the ANOVA model attached to this variable also marks a powerful effect of context, *F*_(1,377)_ = 194.37, *p* < 0.001; η^2^_p_ = 0.34; 1-β = 1.0, with a subjective feeling of time deceleration, further marked in a trail-running context (*M* = 55.69, *SD* = 32.67) compared to an everyday life context without sports practices (*M* = 30.28, *SD* = 29.36).

Similar models were performed with the 2 types of passion as covariates (harmonious passion and obsessive passion). The repeated measures ANOVA performed on the overestimation index revealed a main effect of harmonious passion, *F*_(1,377)_ = 4.37, *p* = 0.04; η^2^_p_ = 0.01; 1-β = 0.55, as well as the main effect of obsessive passion, *F*_(1,377)_ = 5.40, *p* = 0.02; η^2^_p_ = 0.01; 1-β = 0.64. Additional regression analyses revealed that, for both harmonious (*t* = 3.0, β = 0.16, *SE* = 0.15, *p* = 0.002) and obsessive passion (*t* = −2.80, β = 0.14, *SE* = 0.27, *p* = 0.005), an increase in the passion score leads to feelings of greater overestimation of time, regardless of the context. Yet, the ANOVA does not show any main effect of context, nor any interaction effect (*F* < 2, *ps* > 0.05).

The ANOVA performed on the underestimation index also revealed the main effect of obsessive passion, *F*_(1,377)_ = 11.87, *p* = 0.001; η^2^_p_ = 0.03; 1-β = 0.93. It is strange to note that, once again, the greater the obsessive passion, the greater the feeling of the time deceleration (*t* = 2.12, β = 0.11, *SE* = 0.26, *p* = 0.03; η^2^_p_ = 0.01; 1-β = 0.55), as well as a context × obsessive passion interaction effect, *F*_(1,377)_ = 4.28, *p* = 0.04; η^2^_p_ = 0.01; 1-β = 0.54). Additional linear regression analysis of the type of passion on the difference between the sensation of time dilation from everyday life to trail-running context revealed that increasing passion led to increasing time dilation (*t* = −2.73, β = −0.14, *SE* = 0.44, *p* = 0.007; *t* = −2.76, β = −0.14, *SE* = 0.24, *p* = 0.006 for both harmonious and obsessive passion, respectively). In sum, subjects reported experiencing greater time dilation in a trail-running situation. This effect depends on the type of passion, which is more marked as the passion scores increase (either harmonious or obsessive). In addition, we found that an increase in passion scores leads participants to experience more temporal acceleration in their lives, regardless of the context. Incongruously, participants with a high obsessive passion score also do report a higher temporal contraction in their lives, regardless of the context.

## Discussion

The aim of this study was to measure the feeling of the PoT of ultra-trail runners, both within the context of their sports and outside of this context, in a daily life situation. In addition, the objective was to relate these perceptions with the ultra-trail runners' type of passion (harmonious vs. obsessive). The results obtained confirm the first hypothesis according to which the participants would experience a dilation in perceived time in a sports situation, compared to an everyday life situation without physical activity. It also confirmed the second hypothesis stipulating that this distortion would be in favor of time dilation. Indeed, the participants systematically reported feelings of temporal distortions, as well as greater feelings of dilation of time in the context of the ultra-trail compared to the daily life situation. This study, therefore, extends the findings on the effects of physical activity on time perception already obtained in interval length judgments for both short and long durations (for a review, refer to Behm and Carter, 2020). According to PoT studies, the level of happiness felt and cognitive abilities are the two major factors that modulate awareness of time perception (Winkler et al., 2017). The effect of the level of happiness can hardly be conceived as responsible for this effect here, since happiness would have the effect of time contraction (Droit-Volet and Wearden, 2015, 2016; Droit-Volet and Trahanias, 2017; Droit-Volet, 2019; Droit-Volet et al., 2021). Since our results are directed in the opposite way, it would, therefore, be necessary to assume that the runners associate a negative feeling with their practices, which is why it is hard to conceive; otherwise, runners would most likely reduce their engagement in ultra-trails. The most probable hypothesis relates to the influence of attention. Based on previous research, the effect of overestimation in interval duration judgments as a function of the intensity of the exercise has been explained by the state of hyperarousal that physical activity generates and which increases sensory awareness. Although our study was not designed to demonstrate the nature of the effect, it is likely that this mechanism of higher awareness, highlighted by researchers, could be responsible for the effect. Arousal on its own could not predict the lengthening effect, as higher arousal, necessarily linked to physical activity, would have the effect of shortening time passage (Wearden et al., 2014; Droit-Volet and Wearden, 2015, 2016). Thus, the only explanatory variable remaining would be that of attention. A heightened state of awareness, as already put forward in previous studies, would be permitted by the state of hyperactivation generated by the high intensity of running, which ultimately would allow runners to process and store more information (internal and external) than usual during a given time. These increased amounts of information grasped result in the feeling that time is longer than it really is.

For the first time, our study demonstrated that the type of passion can have specific effects on time perception. This is an important result, and even more regarding PoT judgments, as very few studies showed that individual traits can be related to this measure (Martinelli et al., 2021). As hypothesized, higher scores of harmonious passion were related to a greater sensation of time contraction. This could easily be related to social studies showing that higher harmonious scores lead to higher levels of well-being (Carpentier et al., 2012; Moè, 2016). As well-being increases, the feeling of a PoT is accelerated (Droit-Volet and Wearden, 2015, 2016; Droit-Volet and Trahanias, 2017; Droit-Volet, 2019; Droit-Volet et al., 2021). Yet, our fourth hypothesis predicting that obsessive passion scores would be related to greater feelings of time dilation was only partially validated. Indeed, the more the participants had a high obsessive score, the more they reported both feelings of dilated and contracted time. The inconsistency of the results regarding the direction of this result on obsessive passion makes it difficult to explain these effects. Further studies, thus, need to be carried out in order to identify the mechanisms underlying these effects. However, we proposed three different possibilities that could explain our results.

The first explanation could be the lack of compartmentalization between the passionate activity and other activities. Daily non-sportive activities would thus be more permeable, or even non-existent for individuals with high obsessive passion scores, contrarily to high harmonious passion scores. This is also the reason put forward by researchers explaining why participants with an obsessive passion for their work are more likely to develop burnout symptoms (Vallerand et al., 2010). Thus, negative states of sadness or frustration could generate underestimation of time, as previously exposed in the literature (Tipples, 2018; Kent et al., 2019), but the passionate activity could penetrate each of the ancillary activities, thus being able to dilate time perception by a reviviscence phenomenon of the physical activity.

Another possibility would be that of heterogeneity within the delays of non-practice which would affect differently the PoT of the participants with high obsessive passion. If someone assumes that obsessive passion leads to negative feelings that slower the PoT judgment when the participant is not practicing the passion activity, which does not prevent them from being as happy as their harmonious passionate peers during the running practice (or related activity). As a consequence, the phenomenon that PoT is judged longer within individuals with high obsessive passion would only occur later than the practice and thus mainly depends on how long they can ruminate and be frustrated from not being able to practice their activity. It would be interesting to control the delay without practicing in a future study.

Finally, another alternative that could explain that obsessive passion scores predict feelings of speeding up and slowing down of the PoT would be that these individuals would experience timelessness. This explanation could thus help to understand why the participants with high obsessive passion appear as being lost in time. This hypothesis seems particularly relevant since it would also allow us to understand the lack of internal consistency between our measures (α = 0.57). Note that this lack of internal consistency could result from the participants' misunderstanding, but this seems hardly conceivable as similar results were found on the two constructs: “time goes fast” and “the elapsed time is short.” In contrast, the results question the use of the item “Time stands still” as it may be interpreted as something even beyond time dilation. Further experimental investigations are thus needed to better understand what lies behind the confusing sensations of PoT in participants with high obsessive passion scores.

Since our study is, to the best of our knowledge, the first to document the PoT judgment in sports, it would be appropriate to pursue the studies and to further analyze these effects. For example, it would be interesting to study the PoT judgment during the ultra-trail. Indeed, the PoT could oscillate from the beginning to the end of the race, which could, therefore, partly explain why some subjects report both dilations and underestimations of time in sports. Yet, as it stands, this would not explain why the effect would be more pronounced in participants with obsessive passion. Also, someone should analyze whether the duration since the last ultra-trail accentuates the feeling of time distortion. However, this would not explain the way the distortion (e.g., dilation of time judgments) was observed. Finally, it seems crucial to investigate the result suggesting that individuals with an obsessive passion appear to be lost in time and may experience timelessness, as it could reflect that obsessive passion has more intense emotional levels, which could constitute a factor of the vulnerability of health and well-being.

To conclude, this study extends the findings of the previously documented effects of sports practice on the perception of time using interval duration judgments. The results show a dilation of time in a situation of ultra-trail practice compared to a daily situation without physical practice. Paradoxically, accelerating, bustling, and running would, therefore, be a way of slowing down the perception of the PoT. Our study also demonstrated the influence of the type of passion on the perception of PoT. According to our results, participants with high harmonious passion scores showed higher sensations of time dilation, while participants with high obsessive passion scores showed higher sensations of both dilation and contraction of time. These results thus offer a new research avenue on the effect of the type of passion on time perceptions. This is an important perspective because, from a methodological point of view, these results imply taking the type of passion into account in future research.

## Data availability statement

The raw data supporting the conclusions of this article will be made available by the authors, without undue reservation.

## Ethics statement

Ethical review and approval was not required for the study on human participants in accordance with the Local Legislation and Institutional Requirements. The patients/participants provided their written informed consent to participate in this study.

## Author contributions

All authors listed have made a substantial, direct, and intellectual contribution to the work and approved it for publication.
